# A Retrospective Cohort Analysis Shows that Coadministration of Minocycline with Colistin in Critically Ill Patients Is Associated with Reduced Frequency of Acute Renal Failure

**DOI:** 10.1128/AAC.01165-17

**Published:** 2017-12-21

**Authors:** Thomas P. Lodise, Weihong Fan, David C. Griffith, Michael N. Dudley, Katherine A. Sulham

**Affiliations:** a Albany College of Pharmacy and Health Sciences, Albany, New York, USA; b The Medicines Company, Parsippany, New Jersey, USA; c The Medicines Company, San Diego, California, USA

**Keywords:** multidrug resistance, acute renal failure, nephrotoxicity, minocycline, colistin

## Abstract

Nonclinical studies have suggested that oxidative damage, caspase-mediated apoptosis, and inducible nitric oxide synthase levels may be involved in the pathogenesis of colistin (CST)-associated acute renal failure. MIN inhibits caspase 1, caspase 3, and inducible nitric oxide synthase, leading to the hypothesis that coadministration of CST with MIN (CST-MIN) may reduce the incidence of acute renal failure as well as produce additive or synergistic antimicrobial effects. A multicenter retrospective cohort study was conducted using the Premier Research database to examine the impact of CST-MIN on acute renal failure. Inclusion criteria were as follows: age of ≥18 years, intensive care unit admission at CST initiation, primary International Classification of Diseases 9 (ICD-9) diagnosis of pneumonia or sepsis, nondialysis at hospital admission, and discharge between January 2010 and December 2015. ICD-9 code 584.XX or ICD-10 code N17 was used to define acute renal failure. Baseline comparisons, 1:8 propensity score matching, and confirmatory logistic regression analyses were conducted. In total, 4,817 patients received CST and met inclusion criteria; 93 received CST-MIN. Unadjusted frequency of acute renal failure was significantly lower in patients receiving CST-MIN than CST (11.8% versus 23.7%, *P* = 0.007). Similar results were seen in propensity score matching (12.0% versus 22.3%, *P* = 0.031) and logistic regression analyses (odds ratio of 0.403, *P* = 0.006). Mortalities and 30-day readmission rates were similar between groups. The acute renal failure rate was not impacted by prevalence of baseline renal disease. CST-MIN in critically ill patients may reduce CST-associated acute renal failure. Further evaluation of this combination in prospective clinical studies is warranted.

## INTRODUCTION

The prevalence of serious infections due to highly resistant Gram-negative pathogens is increasing worldwide (1–3). Due to the dearth of commercially available antibiotics with reliable microbiologic activity against multidrug-resistant Gram-negative bacteria (MDR-GNB), clinicians have often resorted to using older agents that carry a risk of toxicity, such as colistin (CST), alone or in combination with other agents (4, 5). While long considered an agent of last resort, there has been a substantial increase in CST use over the past decade. Recent studies indicate that it is frequently employed as an early directed therapy in combination with other agents in patients with documented or suspected infections due to MDR-GNB in many parts of the world (6–8).

The occurrence of acute renal failure (ARF) with CST use remains problematic (9). A recent study showed that CST exposures required for a demonstrable antibacterial effect (1-log killing in a neutropenic mouse thigh infection model) are associated with ARF rates of 32% at day 7 and 52% at end of treatment (10). Since the occurrence of ARF appears to be unavoidable with the use of CST at the currently recommended therapeutic dosing regimens, a number of studies have explored the mechanism of polymyxin-induced nephrotoxicity and the potential role of nephroprotective agents to minimize the extent of CST-induced ARF and have produced differing results (11–13).

Minocycline (MIN) is a member of the tetracycline class of antibiotics, with broad-spectrum activity against many Gram-positive and Gram-negative bacteria. It is active against Acinetobacter spp., including MDR strains, and has been increasingly used alone or in combination with other agents, including polymyxins, in the treatment of infections due to these pathogens (14). Data suggest that combining MIN with CST may produce synergistic bactericidal effects (15). MIN also has a number of other nonantimicrobial pharmacological effects, including inhibition of the production of mediators of inflammatory and apoptotic pathways that have been associated with nephrotoxicity due to polymyxins (16). We hypothesized that MIN may have nephroprotective effects in patients when administered in combination with CST (CST-MIN). We used a large, representative hospital database to test the hypothesis that patients in the intensive care unit (ICU) who received CST-MIN have a lower occurrence of ARF than patients who received CST without MIN.

## RESULTS

### Demographic and baseline characteristics.

**FIG 1:**
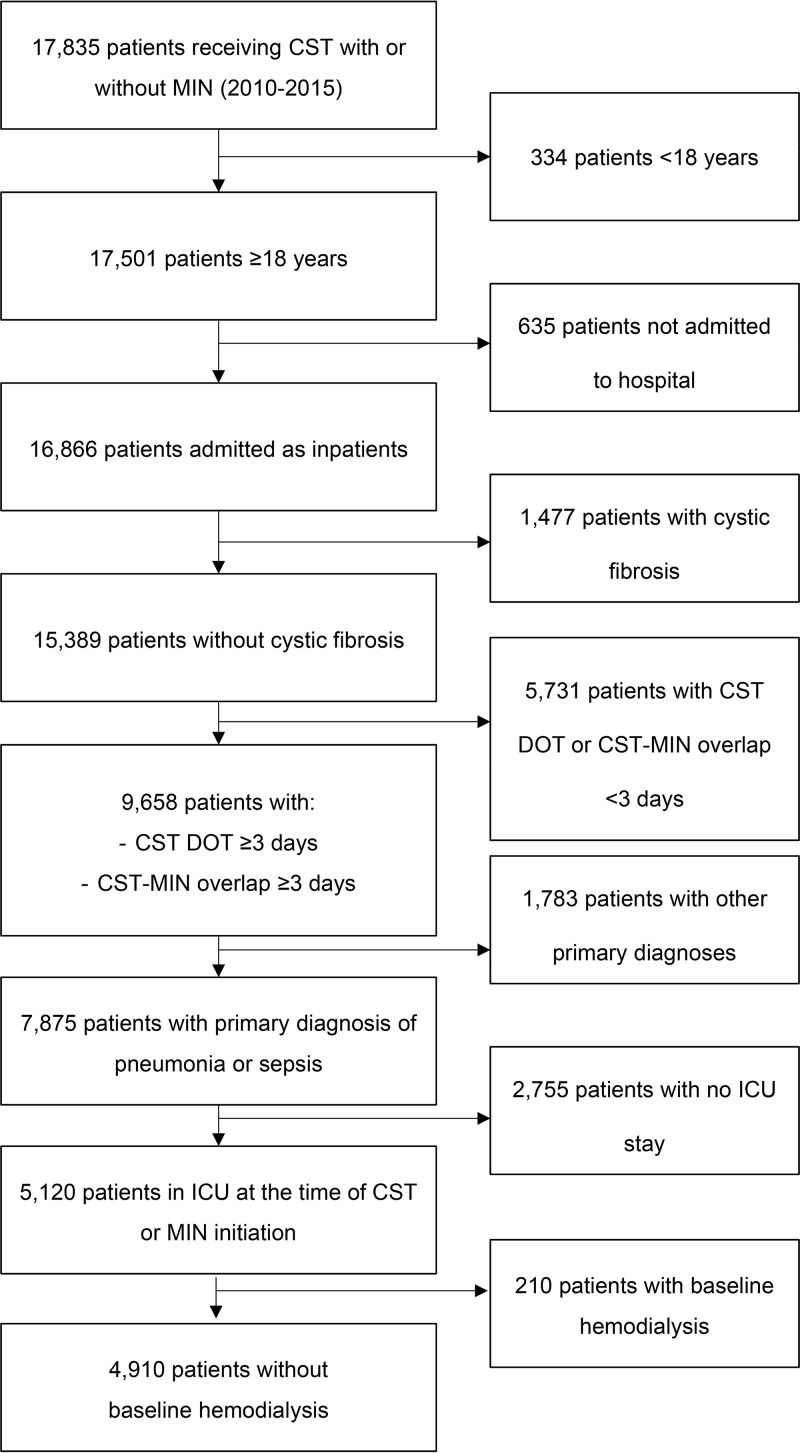
Patient selection flowchart. Abbreviations: CST, colistin; MIN, minocycline; DOT, days of therapy; ICU, intensive care unit.

In this study, 4,910 patients were eligible for analyses (4,817 receiving CST and 93 receiving CST-MIN) (Fig. 1). CST patients were slightly younger than those receiving CST-MIN (mean age, 61.3 ± 16.0 years versus 64.6 ± 16.2 years, *P* = 0.040); other baseline characteristics were similar between the two groups. Mean and median Charlson comorbidity index scores were similar between the groups, though with respect to the individual comorbidities, CST patients had a significantly lower prevalence of baseline chronic renal disease (CRD) (37.1% with CST versus 50.5% with CST-MIN, *P* = 0.008) and HIV/AIDS (0.6% with CST versus 3.2% with CST-MIN, *P* = 0.026) (Table 1). No differences in infection type (pneumonia versus sepsis) or patient distribution by year were found between treatment groups. Use of other medications that could cause ARF (see Table S1 in the supplemental material) was quite frequent in both the CST and CST-MIN groups (98.2% with CST versus 98.9% with CST-MIN, *P* = 1.000) (Table S2); however, it varied with type of medications, particularly the use of contrast media (11.1% with CST versus 33.3% with CST-MIN, *P* < 0.001) and tobramycin (30.3% with CST versus 66.7% with CST-MIN, *P* < 0.001).

**TABLE 1:** Demographic and baseline characteristics^*a*^

Patient variable	Value for:					
	Primary population			PSM population		
	CST (*n* = 4,817)	CST-MIN (*n* = 93)	*P* value^*b*^	CST (*n* = 664)	CST-MIN (*n* = 83)	Absolute standard difference (%)
Age (yr)						
Mean ± SD	61.3 ± 16.0	64.6 ± 16.2	**0.040**	63.6 ± 14.6	63.4 ± 16.0	1.7
Median (Q1, Q3)	63 (52, 73)	66 (57, 77)		65 (55, 75)	65 (56, 74)	
Age ≥ 65 yr, %	45.9	53.8	0.130	51.7	50.6	2.1
Male, %	58.2	48.4	0.058	51.4	50.6	1.5
White, %	58.4	62.4	0.735	63.3	62.7	1.2
Primary payer, %						
Medicare	61.3	63.4	0.598	63.3	61.4	3.7
Medicaid	19.3	16.1		17.2	16.9	0.8
Commercial/managed	14.1	17.2		14.8	18.1	9.0
Other	5.2	3.2		4.8	3.6	6.0
CCI score						
Mean ± SD	3.1 ± 2.3	3.2 ± 2.3	0.519	3.2 ± 2.1	3.2 ± 2.3	2.9
Median (Q1, Q3)	3 (1, 4)	3 (2, 4)		3 (2, 4)	3 (2, 4)	
Charlson comorbidity, %						
Myocardial infarction	8.9	5.4	0.231	6.2	6.0	0.6
CHF	31.9	36.6	0.341	33.6	34.9	2.9
PVD	11.2	9.7	0.638	9.2	9.6	1.5
Cerebrovascular disease	10.9	6.5	0.168	9.8	7.2	9.2
Dementia	3.6	3.2	1.000	4.2	3.6	3.1
COPD	37.9	35.5	0.639	36.4	36.1	0.6
Rheumatic disease	3.2	4.3	0.567	3.6	4.8	6.0
Peptic ulcer disease	2.9	2.2	1.000	2.1	1.2	7.1
Mild liver disease	8.2	3.2	0.085	4.7	3.6	5.3
Diabetes without complication	36.4	33.3	0.549	36.6	34.9	3.5
Diabetes with complication	7.0	3.2	0.213	3.2	3.6	2.5
Paraplegia and hemiplegia	14.9	14.0	0.795	15.8	15.7	0.4
Chronic renal disease	37.1	50.5	**0.008**	47.0	47.0	0.0
Cancer	7.1	7.5	0.868	7.7	8.4	2.8
Moderate/severe liver disease	1.9	2.2	0.699	2.3	1.2	8.1
Metastatic carcinoma	2.6	1.1	0.733	1.8	1.2	4.9
HIV/AIDS	0.6	3.2	**0.026**	1.1	3.2	8.5
Region, %						
Midwest	19.1	8.6	**<0.001**	9.6	9.6	0.0
Northeast	16.0	0.0		0.0	0.0	0.0
South	55.1	90.3		89.2	89.2	0.0
West	9.8	1.1		1.2	1.2	0.0
Urban hospital, %	95.1	100.0	**0.023**	100.0	100.0	0.0
Teaching hospital, %	52.6	38.7	**0.008**	44.3	39.8	9.2
Hospital bed size, %						
0–299	26.5	33.3	0.298	27.5	30.1	6.0
300–499	40.4	34.4		36.3	37.3	2.2
>500	33.0	32.3		36.3	32.5	7.9
Infection type, %						
Pneumonia	74.3	68.8	0.228	70.5	71.1	1.3
Sepsis	85.6	88.2	0.479	88.3	86.7	4.6

a Abbreviations: PSM, propensity score matching; CST, colistin; CST-MIN, colistin-minocycline; CCI, Charlson comorbidity index; CHF, congestive heart failure; PVD, peripheral vascular disease; COPD, chronic obstructive pulmonary disease.

b *P* values in bold indicate a significance difference.

Hospital characteristics varied between groups, with CST-MIN patients more likely to be treated at urban hospitals (95.1% with CST versus 100.0% with CST-MIN, *P* = 0.023) and CST patients more likely to be treated at teaching hospitals (52.6% with CST versus 38.7% with CST-MIN, *P* = 0.008) (Table 1).

### Study drug dosing and administration.

Patients in the CST-MIN group received significantly more days of CST therapy than those receiving CST alone (mean, 10.0 days with CST versus 12.7 days with CST-MIN, *P* = 0.009), although the total numbers of vials of CST were similar between groups (mean, 21.4 vials with CST versus 22.8 vials with CST-MIN, *P* = 0.428). Distribution of the number of daily vials received was similar between the treatment groups, including patients with and without CRD (Table S3). The mean (median) number of days of study drug overlap in the CST-MIN group was 7.2 (6) (Table 2). In the CST-MIN group, MIN was initiated prior to CST therapy in 18 (19.4%) cases, after CST in 42 (45.2%) cases, and concomitantly with CST in 33 (35.5%) cases. In the 42 patients in whom MIN was started after CST, CST use was initiated a median of 4 days before the initiation of MIN, and the median study drug overlap in this subset of patients was 6 days.

**TABLE 2:** Study drug dosing and administration^*a*^

Patient variable	Value for patients treated with:		*P* value^*b*^
	CST (*n* = 4,817)	CST-MIN (*n* = 93)	
No. of days on MIN			
Mean ± SD	NA	10.1 ± 7.6	
Median (Q1, Q3)	NA	8 (5, 14)	
Total no. of vials of MIN			
Mean ± SD	NA	21.5 ± 18.6	
Median (Q1, Q3)	NA	16 (10, 29)	
Daily no. of vials of MIN, % of patients			
2 vials	NA	88.2	
3 vials	NA	8.6	
4 vials	NA	3.2	
No. of days on CST			
Mean ± SD	10.0 ± 8.3	12.7 ± 10.4	**0.009**
Median (Q1, Q3)	8 (5, 12)	9 (6, 15)	
Total no. of vials of CST			
Mean ± SD	21.4 ± 23.2	22.8 ± 18.9	0.428
Median (Q1, Q3)	15 (9, 27)	17 (9, 30)	
Daily no. of vials of CST,^*c*^ % of patients			
1 vial	22.3	35.5	0.058
2 vials	54.1	51.6	
3 vials	15.6	9.7	
≥4 vials	8.0	3.2	
No. of days of treatment overlap			
Mean ± SD	NA	7.2 ± 4.3	
Median (Q1, Q3)	NA	6 (4, 9)	
No. of days from admission to initiation of study drug			
Mean ± SD	14.6 ± 20.0	14.3 ± 23.2	0.069
Median (Q1, Q3)	8 (4, 18)	5 (2, 17)	
Sequence of CST-MIN administration, % of patients			
MIN initiated prior to CST	NA	19.4	
MIN and CST initiated on the same day	NA	35.5	
MIN initiated after CST	NA	45.2	

a Abbreviations: CST, colistin; CST-MIN, colistin-minocycline; MIN, minocycline; NA, not applicable.

b *P* values in bold indicate a significance difference.

c One vial of colistin = 150 mg colistin-based activity (CBA).

### Unadjusted outcomes.

In the unadjusted analysis, patients receiving CST-MIN were significantly less likely to experience ARF than those receiving CST (23.7% with CST versus 11.8% with CST-MIN, *P* = 0.007; unadjusted odds ratio [OR], 0.431; 95% confidence interval [CI], 0.229, 0.812). Mortality and 30-day all-cause readmission rates were similar between groups (Table 3). ARF rates were numerically higher in patients with baseline CRD receiving CST than in those receiving CST-MIN (22.3% with CST versus 10.6% with CST-MIN, *P* = 0.058); similar results were seen in those without baseline CRD (ARF rate, 24.6% versus 13.0% in patients receiving CST and CST-MIN, respectively, *P* = 0.070).

**TABLE 3:** Unadjusted and adjusted outcomes in patients treated with CST or CST-MIN^*a*^

Parameter^*b*^	Value for patients treated with:		OR (95% CI)	*P* value^*c*^	C statistic
	CST	CST-MIN			
Unadjusted outcomes, % of patients	*n* = 4,817	*n* = 93			
ARF	23.7	11.8	0.431 (0.229, 0.812)	0.007	
Mortality	29.5	31.2	1.081 (0.694, 1.683)	0.731	
30-day readmission	26.6	31.3	1.256 (0.736, 2.142)	0.402	
PSM (1:8 matching), % of patients	*n* = 664	*n* = 83			
ARF	22.3	12.0	0.478 (0.241, 0.948)	0.031	
Mortality	28.9	32.5	1.185 (0.727, 1.933)	0.495	
30-day readmission	29.9	30.4	1.023 (0.560, 1.870)	0.940	
Logistic regression model	*n* = 4,817	*n* = 93			
ARF			0.403 (0.211, 0.770)	**0.006**	0.673
Mortality			1.031 (0.642, 1.656)	0.898	0.714
30-day readmission			1.139 (0.664, 1.954)	0.636	0.577

a Abbreviations: PSM, propensity score matching; OR, odds ratio; CI, confidence interval; ARF, acute renal failure.

b Thirty-day readmission was estimated based on the patients who survived from index hospitalization.

c *P* values in bold indicate a significance difference.

We further evaluated if the number of days of treatment with CST alone versus the number of days with CST-MIN was associated with different rates of ARF. The frequency of ARF increased with the number of days on CST (from 20.1% with 3 to 5 days of CST treatment to 28.7% with ≥14 days of CST treatment). A similar increase in ARF, though of a smaller magnitude, was observed with an increasing number of days of CST-MIN coadministration overlap (from 9.5% with a 3- to 5-day study drug overlap to 15.4% with ≥14 days of study drug overlap) (Table 4). No differences were seen in ARF rates by timing of MIN administration relative to CST administration (e.g., initiation of MIN prior to, at the same time, or after initiation of CST) (data not shown).

**TABLE 4:** Effect of number of days on CST or CST-MIN on incidence of ARF^*a*^

Duration of CST treatment (days)	Frequency (%) of ARF in patients on CST	Duration of CST-MIN coadministration^*b*^ (days)	Frequency (%) of ARF in patients on CST-MIN	*P* value
3–5	305/1,517 (20.1)	3–5	2/21 (9.5)	0.284
6–8	266/1,179 (22.6)	6–8	2/19 (10.5)	0.275
9–13	280/1,102 (25.4)	9–13	3/27 (11.1)	0.115
≥14	292/1,019 (28.7)	≥14	4/26 (15.4)	0.186

a Abbreviations: ARF, acute renal failure; CST, colistin; CST-MIN, colistin-minocycline.

b Duration of overlap of CST and MIN; duration of either or both study drugs individually may be longer than the duration of overlap.

### Adjusted outcomes.

Also in this study, 83/93 (89.3%) CST-MIN patients were matched 1:8 with 664 patients receiving CST. Propensity score matching (PSM) results were consistent with the unadjusted analysis: patients receiving CST-MIN were less likely to experience ARF than those receiving CST (22.3% with CST versus 12.0% with CST-MIN; OR, 0.478; *P* = 0.031) (Table 3). Confirmatory logistic regression found an OR of 0.403 for ARF in CST-MIN versus CST patients (*P* = 0.006). Both the PSM method and the conventional logistic regression modeling technique confirmed that mortality and 30-day readmission rates remained similar between the groups.

### Attributable costs and LOS associated with ARF.

In addition, 1,068 patients with ARF were matched 1:1 to patients without ARF. Attributable costs and length of stay (LOS) associated with ARF were $13,931 (*P* < 0.001) and 1.81 days (*P* = 0.037), respectively. Similar results were seen with confirmatory logistic regression; ARF was associated with an incremental cost increase of $10,837 and an additional 3.02 days in LOS (*P* < 0.001 for both) (Table 5).

**TABLE 5:** Attributable cost and LOS associated with ARF^*a*^

Parameter^*b*^	Value for patients		Difference (95% CI)	*P* value	C statistic
	Without ARF	With ARF			
PSM	*n* = 1,068	*n* = 1,068			
Incremental costs of ARF ($)	60,784	74,715	13,931 (8,251, 19,611)	<0.001	
Incremental LOS of ARF (mean [median] no. of days)	20.34 (14)	22.14 (16)	1.81 (0.11 3.50)	0.037	
Logistic regression model	*n* = 3,756	*n* = 1,154			
Incremental costs of ARF ($)			10,837 (8,039, 13,635)	<0.001	0.667
Incremental LOS of ARF (mean [median] no. of days)			3.02 (1.80, 4.23)	<0.001	0.128

a Abbreviations: ARF, acute renal failure; PSM, propensity score matching; CI, confidence interval; LOS, length of stay.

b Incremental costs and LOS were calculated post-study drug initiation.

## DISCUSSION

The lack of availability of new agents with demonstrated clinical efficacy against infections due to MDR-GNB has resulted in an increased use of older, “last resort” antibiotics like CST. Although studies clearly demonstrate that the use of CST results in an increased incidence of ARF, particularly in critically ill patients, clinicians have few available options for treating patients with these life-threatening infections.

CST alone or in combination with other antimicrobial agents has been increasingly used to manage infections due to MDR-GNB; however, nephrotoxicity may occur in up to 40% of patients treated with CST or other polymyxins (9). The mechanism of polymyxin-induced nephrotoxicity is associated with uptake into kidney tissue and membrane effects on proximal tubular cells. Recent studies with polymyxin B in human and rat renal proximal tubular cell lines demonstrated concentration- and time-dependent apoptosis. DNA breakage associated with certain apoptotic pathways, including those activated by caspases, was also observed (11). *In vivo* studies in a rat model demonstrated that the pathogenesis of CST-induced nephrotoxicity was associated with increases in markers for inflammation, oxidative damage, and apoptosis, including involvement of caspase 1, calpain 1, and inducible nitric oxide synthase (iNOS) pathways (17). Consistent with the involvement of these pathways in polymyxin-induced nephrotoxicity, studies have shown that damage to kidney cells can be prevented through the use of antioxidant agents (12, 13, 17).

MIN is a tetracycline agent with broad-spectrum activity against many bacteria, including Acinetobacter spp. Microbiological studies have shown *in vitro* synergism against Acinetobacter, including isolates resistant to MIN or CST (14, 18). In addition to the potential microbiological and therapeutic benefit of the combination, we hypothesized that the nonantimicrobial effects of MIN might mitigate CST-mediated nephrotoxicity. MIN demonstrates antioxidant properties, inhibition of caspase 1 and caspase 3 activation, inhibition of iNOS, and enhancement of Bcl-2-derived effects (19–21). All of these properties could be useful in reducing apoptosis and oxidative damage associated with polymyxins.

In view of the potential nephroprotective effects of MIN in patients treated with polymyxins, we conducted a retrospective cohort study to assess if critically ill patients who received CST-MIN had lower rates of ARF than those who received CST without MIN. Similar to other studies, the overall occurrence of ARF in patients who received CST without MIN was 23.7%, and rates of ARF increased as a function of CST duration. In contrast, the overall occurrence of ARF among patients who received CST-MIN was 11.8%, and occurrence of ARF increased only modestly with prolonged CST therapy, with a consistent reduction of approximately 50% in the CST-MIN cohort compared with that in the CST cohort, regardless of therapy duration. Interestingly, the rate of ARF observed in patients who received CST-MIN is comparable to the incidence of ARF typically reported in patients with infections in the ICU who do not receive well-described nephrotoxic antibiotics (22–26).

The disparity in ARF rates between the groups does not appear to be due to confounding or prescribing bias, as patients who received CST-MIN were typically older, had a higher prevalence of baseline CRD, and were more likely to receive other agents that could result in ARF than patients who received CST without MIN. The consistency in findings in the stratified, propensity-score-matched, and multivariate analyses further suggest that results were not severely distorted by other conditions or discordant baseline characteristics between treatment groups. Duration of CST therapy, a major driver of CST-associated acute kidney failure (10), was also found to be longer among patients who received CST-MIN than among those who received CST, further minimizing the likelihood of biased results due to systemic errors. In contrast, the weighted mean number of daily vials received was higher among CST patients, and this was true for patients both with and without CRD. However, distribution of the number of daily vials administered, which is a better marker of what was actually administered, was similar between CST groups. Unfortunately, data on the actual CST dose received was not available, as Premier Research reports only the number of vials administered. If one considers the number of vials received as a surrogate for the actual daily dose, CST patients, on average, received only 0.25 more vials per day than the CST-MIN patients. This equates to ∼50 mg more of colistin-based activity per day in the CST group than in the CST-MIN group. Given the modest daily vial difference between groups and substantial (∼12) interpatient variability in the plasma colistin concentrations associated with colistimethate, it is unlikely that the differences in number of daily vials substantially contributed to the differences in ARF finding between groups. To further test that these nephroprotective effects were unique to MIN relative to other tetracyclines, we performed a *post hoc* analysis in patients who received CST without tigecycline (TIG) in comparison to those receiving CST in combination with tigecycline (CST-TIG), using the methodology described herein. Rates of ARF were comparable between patients who received CST and those receiving CST-TIG (unadjusted ARF rate, 22.0% with CST versus 27.6% with CST-TIG) (data not shown). Collectively, the findings from this analysis support the hypothesis that the use of CST-MIN may reduce CST-induced ARF rates.

These findings may have important implications for clinical practice if future studies substantiate the results of this pilot study. Although no meaningful differences in outcomes were noted between comparison groups, patients who experienced ARF had an associated approximate 3-day increase in hospital LOS and more than $10,000 in excess hospital costs. These findings are consistent with other published studies, which clearly demonstrate that the occurrence of ARF, regardless of cause, results in considerable increases in morbidity, mortality, and health care expenditures (27, 28).

Several limitations should be noted when interpreting these findings. This was a retrospective, observational study and is subject to all the limitations associated with this study design. Comorbidity data and diagnoses are coded using ICD-9 and ICD-10 codes, which may not be consistent across hospitals. Of the approximate 5,000 patients who received CST either alone or in combination, only 93 received CST-MIN; therefore, results may be imprecise, especially from the stratified analyses. The Premier Research database does not contain detailed laboratory results; therefore, occurrence of ARF was based on diagnosis codes. While use of serum creatinine data to determine ARF would have been preferred, our approach is associated with high specificity and positive predictive value (29, 30). We further reduced the chance of coding bias by using a single ICD code indicative of ARF rather than a group of several codes that may fall under the broader definition of acute kidney injury but would be subject to higher rates of misclassification. Independent nondifferential outcome disease misclassification with perfect specificity would not bias the risk-ratio estimate and would only downwardly bias the absolute magnitude of the risk-difference estimate by a factor equal to the false-negative probability. Therefore, more attention should be placed on the relative risk contrasts between treatment groups rather than the absolute differences.

Microbiology data were available only in a small subset of patients: 919 (17.9%) patients had microbiology data available (918 CST patients, 1 CST-MIN patient). The most commonly reported pathogens included Enterobacteriaceae (*n* = 533, 56.8%), Pseudomonas aeruginosa (*n* = 425, 46.3%), and Acinetobacter baumannii (*n* = 400, 43.5%). It is unclear if observed differences in ARF rates between treatment groups are biased due to differences in the invading pathogen. However, the study was restricted to patients in the ICU, and there were no notable differences in collected baseline characteristics and CST therapy to suggest that findings were distorted due to systemic errors. We did not include polymyxin B in our analyses, because too few patients received it in combination with MIN in the master data set (*n* = 4); however, rates of ARF among ICU patients who received polymyxin B were comparable to those observed in patients with CST (24.1% [106/439] versus 23.7% [1,143/4,817], respectively). In addition, detailed weight/body mass index and dosing information is not available in the Premier Research database; drug use is reported as vials only, and therefore we did not attempt to convert to colistin-based activity.

In conclusion, the findings from this large, representative, retrospective multicenter cohort analysis suggest that coadministration of MIN with CST in critically ill patients may reduce the occurrence of CST-associated ARF. At a minimum, these findings should be evaluated in prospective clinical studies that include collection of detailed laboratory (including serum creatinine concentrations), microbiologic, treatment, and outcome data. If these findings are validated, we may be able to vastly improve the care of difficult-to-treat patients with infections due to MDR-GNB by combining MIN with CST.

## MATERIALS AND METHODS

We conducted a multicenter retrospective cohort study using the Premier Research database to examine the impact of CST-MIN on ARF. Because this study used already existing Health Insurance Portability and Accountability Act (HIPAA)-compliant fully deidentified data, it was exempt from Institutional Review Board review.

### Data source.

The Premier Research database is an electronic laboratory, pharmacy, and billing data repository that contains data from more than 600 U.S. hospitals, comprising nearly 20% of all hospitalizations nationwide. In addition to patient age, gender, race/ethnicity, insurance information, principal and secondary diagnoses, and procedures, the database contains a date-stamped log of all medications, laboratory tests, and diagnostic and therapeutic services charged to the patient or their insurer. The database also includes total and individual component costs. In addition, 176 contributing institutions submit microbiology data, including pathogen and susceptibility information.

### Study population.

Patients were eligible for study inclusion if they were ≥18 years of age, hospitalized with a primary diagnosis of pneumonia or sepsis (or secondary diagnosis in the setting of respiratory failure), and discharged between 1 January 2010 and 31 December 31 2015. Pneumonia was identified by the principal diagnosis International Classification of Diseases (ICD)-9-CM codes 480 to 486 (ICD-10-CM codes J12 to J18) or by respiratory failure code 518.81 or 518.84 (ICD-10-CM code J96.0X or J96.2X) with pneumonia as a secondary diagnosis. Sepsis was identified by the principal diagnosis code 038, 038.9, 790.7, 995.91, 995.92, or 785.52 (ICD-10-CM code A41, R78.81, R65.1X, or R65.2X) or by respiratory failure codes with sepsis as a secondary diagnosis (19, 31–33). Patients must have had an ICU stay during study drug administration and must have received a minimum of 3 days of intravenous CST with or without coadministration of intravenous MIN. In the CST-MIN cohort, study drug overlap must have occurred for at least 3 days. Patients in either cohort may have received additional antibiotics at any time during their hospitalization. Patients with a diagnosis of cystic fibrosis or with hemodialysis that occurred in the month immediately prior to the admission date of the hospitalization of interest were excluded from the analysis.

### Data elements and outcomes of interest.

The primary outcome of interest was frequency of ARF, defined as ICD-9 codes of 584.XX or ICD-10 codes of N17.XX (29). Secondary outcomes included in-hospital mortality and 30-day hospital readmission. Baseline characteristics for analysis included demographic characteristics, hospital characteristics, patient comorbidities, index infection, hospital LOS prior to study drug initiation, source of admission, receipt of other medications that could cause ARF (including amikacin, gentamicin, contrast media, and others) (see Table S1 in the supplemental material), study drug dose, and duration of treatment. The incremental costs and LOS attributed to ARF were also examined.

### Statistical methods.

The primary objective was to examine the frequency of ARF among patients who received CST compared with that of patients who received CST-MIN. Baseline comparisons between patients receiving CST versus CST-MIN were conducted. Discrete data were reported as frequencies and percentages; continuous data were reported as means, standard deviations (SD), medians, and interquartile ranges. Differences between treatment groups were tested using the Mann-Whitney U test for continuous variables and the chi-square or Fisher's exact test (for cell counts of <5) for categorical variables. ARF frequency was examined in the overall study cohort and stratified by duration of CST use, duration of study drug coadministration, and presence of baseline CRD.

PSM was conducted in cases where each CST-MIN patient was matched to 8 CST patients using nearest-neighbor matching without replacement. Propensity scores were matched using a caliper width 0.2 logit of the standard deviation (34), and exact matches were performed on CRD status and Census Bureau region. We assessed the success of PSM by examining standardized differences in the baseline variables between the matched CST-MIN and CST groups. It has been suggested that a standardized difference of >10% represents the threshold for an important imbalance in a given confounder between treatment groups (35). If the standardized difference was >10%, we reestimated the variables in the logistic regression model and repeated this process until all standardized differences were <10%. Variables included in the regression were age, gender, race (white or nonwhite), diagnosis (pneumonia or sepsis), use of meropenem (yes/no) or tigecycline (yes/no), discharge year, hospital bed size, region, payor type, 17 individual Charlson comorbidities, use of other medications that could cause ARF (yes/no), LOS prior to initiation of CST or MIN, mechanical ventilation use (yes/no), and the number of vials of colistin received. A confirmatory logistic regression analysis was conducted on the entire study population to confirm the results obtained from the PSM-derived subpopulation.

Incremental costs and attributable LOS associated with ARF were explored using PSM with a 1:1 matching ratio (patients with ARF versus those without ARF, regardless of treatment group) and confirmatory conventional regression models.

All tests were two-tailed, and a *P* value of <0.05 was deemed *a priori* to represent statistical significance. Given the nature of exploratory analysis, no adjustment was made for multiple comparisons. All data and analyses were performed using SAS version 9.2 (SAS Institute Inc., Cary, NC).

## Supplementary Material

Supplemental material

## References

[1] Kaye KS, Pogue JM 2015 Infections caused by resistant gram-negative bacteria: epidemiology and management. Pharmacotherapy 35:949–962. doi:10.1002/phar.1636. 26497481

[2] Vincent JL, Rello J, Marshall J, Silva E, Anzueto A, Martin CD, Moreno R, Lipman J, Gomersall C, Sakr Y, Reinhart K, EPIC II Group of Investigators. 2009 International study of the prevalence and outcomes of infection in intensive care units. JAMA 302:2323–2329. doi:10.1001/jama.2009.1754. 19952319

[3] Centers for Disease Control and Prevention. 2013 Antibiotic resistance threats in the United States, 2013. Available at http://www.cdc.gov/drugresistance/threat-report-2013/pdf/ar-threats-2013-508.pdf Accessed 23 February 2017.

[4] Bassetti M, Peghin M, Pecori D 2016 The management of multidrug-resistant Enterobacteriaceae. Curr Opin Infect Dis 29:583–594. doi:10.1097/QCO.0000000000000314. 27584587

[5] Izadpanah M, Khalili H 2015 Antibiotic regimens for treatment of infections due to multidrug-resistant Gram-negative pathogens: an evidence-based literature review. J Res Pharm Pract 4:105–114. doi:10.4103/2279-042X.162360. 26312249 PMC4548428

[6] Parchem NL, Bauer KA, Cook CH, Mangino JE, Jones CD, Porter K, Murphy CV 2016 Colistin combination therapy improves microbiologic cure in critically ill patients with multi-drug resistant gram-negative pneumonia. Eur J Clin Microbiol Infect Dis 35:1433–1439. doi:10.1007/s10096-016-2681-1. 27230510

[7] Cheng A, Chuang YC, Sun HY, Sheng WH, Yang CJ, Liao CH, Hsueh PR, Yang JL, Shen NJ, Wang JT, Hung CC, Chen YC, Chang SC 2015 Excess mortality associated with colistin-tigecycline compared with colistin-carbapenem combination therapy for extensively drug-resistant *Acinetobacter baumannii* bacteremia: a multicenter prospective observational study. Crit Care Med 43:1194–1204. doi:10.1097/CCM.0000000000000933. 25793437

[8] Hachem RY, Chemaly RF, Ahmar CA, Jiang Y, Boktour MR, Rjaili GA, Bodey GP, Raad II 2007 Colistin is effective in treatment of infections caused by multidrug-resistant *Pseudomonas aeruginosa* in cancer patients. Antimicrob Agents Chemother 51:1905–1911. doi:10.1128/AAC.01015-06. 17387153 PMC1891378

[9] Nation RL, Velkov T, Li J 2014 Colistin and polymyxin B: peas in a pod, or chalk and cheese? Clin Infect Dis 59:88–94. doi:10.1093/cid/ciu213. 24700659 PMC4305129

[10] Sorlí L, Luque S, Grau S, Berenguer N, Segura C, Montero MM, Alvarez-Lerma F, Knobel H, Benito N, Horcajada JP 2013 Trough colistin plasma level is an independent risk factor for nephrotoxicity: a prospective observational cohort study. BMC Infect Dis 13:380. doi:10.1186/1471-2334-13-380. 23957376 PMC3765824

[11] Azad MA, Finnin BA, Poudyal A, Davis K, Li J, Nation RL, Velkov T, Li J 2013 Polymyxin B induces apoptosis in kidney proximal tubular cells. Antimicrob Agents Chemother 57:4329–4335. doi:10.1128/AAC.02587-12. 23796937 PMC3754291

[12] Hatipoglu M, Turhan V 2016 Ascorbic acid may be seen as a nephroprotective agent in the prevention of colistin-induced nephrotoxicity. Clin Infect Dis 62:1053–1054. doi:10.1093/cid/ciw018. 26797208

[13] Sirijatuphat R, Limmahakhun S, Sirivatanauksorn V, Nation RL, Li J, Thamlikitkul V 2015 Preliminary clinical study of the effect of ascorbic acid on colistin-associated nephrotoxicity. Antimicrob Agents Chemother 59:3224–3232. doi:10.1128/AAC.00280-15. 25801556 PMC4432219

[14] Greig SL, Scott LJ 2016 Intravenous minocycline: a review in *Acinetobacter* infections. Drugs 76:1467–1476. doi:10.1007/s40265-016-0636-6. 27573640

[15] Liang W, Liu XF, Huang J, Zhu DM, Li J, Zhang J 2011 Activities of colistin- and minocycline-based combinations against extensive drug resistant *Acinetobacter baumannii* isolates from intensive care unit patients. BMC Infect Dis 11:109. doi:10.1186/1471-2334-11-109. 21521536 PMC3098177

[16] Garrido-Mesa N, Zarzuelo A, Galvez J 2013 What is behind the non-antibiotic properties of minocycline? Pharmacol Res 67:18–30. doi:10.1016/j.phrs.2012.10.006. 23085382

[17] Ozkan G, Ulusoy S, Orem A, Alkanat M, Mungan S, Yulug E, Yucesan FB 2013 How does colistin-induced nephropathy develop and can it be treated? Antimicrob Agents Chemother 57:3463–3469. doi:10.1128/AAC.00343-13. 23629704 PMC3719728

[18] Lomovskaya O, Nelson KJ, Rubio-Aparicio D, Sun D, Griffith DC, Dudley MN Minocycline activity is enhanced by polymyxin B in *tetB*-containing isolates of *Acinetobacter baumannii*, abstr 2041. ID Week, 26 to 30 October 2016.

[19] Rothberg MB, Pekow PS, Priya A, Zilberberg MD, Belforti R, Skiest D, Lagu T, Higgins TL, Lindenauer PK 2014 Using highly detailed administrative data to predict pneumonia mortality. PLoS One 9:e87382. doi:10.1371/journal.pone.0087382. 24498090 PMC3909106

[20] Chen M, Ona VO, Li M, Ferrante RJ, Fink KB, Zhu S, Bian J, Guo L, Farrell LA, Hersch SM, Hobbs W, Vonsattel JP, Cha JH, Friedlander RM 2000 Minocycline inhibits caspase-1 and caspase-3 expression and delays mortality in a transgenic mouse model of Huntington disease. Nat Med 6:797–801. doi:10.1038/77528. 10888929

[21] Scarabelli TM, Stephanou A, Pasini E, Gitti G, Townsend P, Lawrence K, Chen-Scarabelli C, Saravolatz L, Latchman D, Knight R, Gardin J 2004 Minocycline inhibits caspase activation and reactivation, increases the ratio of XIAP to smac/DIABLO, and reduces the mitochondrial leakage of cytochrome C and smac/DIABLO. J Am Coll Cardiol 43:865–874. doi:10.1016/j.jacc.2003.09.050. 14998631

[22] Case J, Khan S, Khalid R, Khan A 2013 Epidemiology of acute kidney injury in the intensive care unit. Crit Care Res Pract 2013:479730. 23573420 10.1155/2013/479730 PMC3618922

[23] Santos PR, Monteiro DL 2015 Acute kidney injury in an intensive care unit of a general hospital with emergency room specializing in trauma: an observational prospective study. BMC Nephrol 16:30. doi:10.1186/s12882-015-0026-4. 25885883 PMC4377071

[24] Wang HE, Muntner P, Chertow GM, Warnock DG 2012 Acute kidney injury and mortality in hospitalized patients. Am J Nephrol 35:349–355. doi:10.1159/000337487. 22473149 PMC3362180

[25] Lodise TP, Lomaestro B, Graves J, Drusano GL 2008 Larger vancomycin doses (at least four grams per day) are associated with an increased incidence of nephrotoxicity. Antimicrob Agents Chemother 52:1330–1336. doi:10.1128/AAC.01602-07. 18227177 PMC2292536

[26] Wunderink RG, Niederman MS, Kollef MH, Shorr AF, Kunkel MJ, Baruch A, McGee WT, Reisman A, Chastre J 2012 Linezolid in methicillin-resistant *Staphylococcus aureus* nosocomial pneumonia: a randomized, controlled study. Clin Infect Dis 54:621–629. doi:10.1093/cid/cir895. 22247123

[27] Chertow GM, Burdick E, Honour M, Bonventre JV, Bates DW 2005 Acute kidney injury, mortality, length of stay, and costs in hospitalized patients. J Am Soc Nephrol 16:3365–3370. doi:10.1681/ASN.2004090740. 16177006

[28] Reynolds MR, Cohen DJ, Kugelmass AD, Brown PP, Becker ER, Culler SD, Simon AW 2006 The frequency and incremental cost of major complications among Medicare beneficiaries receiving implantable cardioverter-defibrillators. J Am Coll Cardiol 47:2493–2497. doi:10.1016/j.jacc.2006.02.049. 16781379 PMC1800827

[29] Waikar SS, Wald R, Chertow GM, Curhan GC, Winkelmayer WC, Liangos O, Sosa MA, Jaber BL 2006 Validity of International Classification of Diseases, ninth revision, clinical modification codes for acute renal failure. J Am Soc Nephrol 17:1688–1694. doi:10.1681/ASN.2006010073. 16641149

[30] Patel UD, Hardy NC, Smith DH, et al. 2013 Validation of acute kidney injury cases in the mini-sentinel distributed database, September 20, 2013. Available at https://www.sentinelinitiative.org/sites/default/files/Drugs/Assessments/Mini-Sentinel_Validation-of-Acute-Kidney-Injury-Cases.pdf. Accessed 23 February 2017.

[31] Rothberg MB, Haessler S, Lagu T, Lindenauer PK, Pekow PS, Priya A, Skiest D, Zilberberg MD 2014 Outcomes of patients with healthcare-associated pneumonia: worse disease or sicker patients? Infect Control Hosp Epidemiol 35(Suppl 3):S107–S115. doi:10.1086/677829. 25222889 PMC4559081

[32] Rothberg MB, Zilberberg MD, Pekow PS, Priya A, Haessler S, Belforti R, Skiest D, Lagu T, Higgins TL, Lindenauer PK 2015 Association of guideline-based antimicrobial therapy and outcomes in healthcare-associated pneumonia. J Antimicrob Chemother 70:1573–1579. doi:10.1093/jac/dku533. 25558075 PMC4398467

[33] Martin GS, Mannino DM, Eaton S, Moss M 2003 The epidemiology of sepsis in the United States from 1979 through 2000. N Engl J Med 348:1546–1554. doi:10.1056/NEJMoa022139. 12700374

[34] Austin PC 2011 Optimal caliper widths for propensity-score matching when estimating differences in means and differences in proportions in observational studies. Pharm Stat 10:150–161. doi:10.1002/pst.433. 20925139 PMC3120982

[35] Normand ST, Landrum MB, Guadagnoli E, Ayanian JZ, Ryan TJ, Cleary PD, McNeil BJ 2001 Validating recommendations for coronary angiography following acute myocardial infarction in the elderly: a matched analysis using propensity scores. J Clin Epidemiol 54:387–398. doi:10.1016/S0895-4356(00)00321-8. 11297888

