# Supplementary material for: A Retrospective Cohort Analysis Shows that Coadministration of Minocycline with Colistin in Critically Ill Patients Is Associated with Reduced Frequency of Acute Renal Failure
Source: Antimicrob Agents Chemother. 2017 Dec 21;62(1):e01165-17. doi: 10.1128/AAC.01165-17 (PMC5740356; doi:10.1128/AAC.01165-17)
Supplement: Supplemental material [file supp_62_1_e01165-17__index.html]

A Retrospective Cohort Analysis Shows that Coadministration of Minocycline with Colistin in Critically Ill Patients Is Associated with Reduced Frequency of Acute Renal Failure — Supplemental material 

# A Retrospective Cohort Analysis Shows that Coadministration of Minocycline with Colistin in Critically Ill Patients Is Associated with Reduced Frequency of Acute Renal Failure

## Supplemental material

- Supplemental file 1 -

  Supplemental Tables S1 to S3

  PDF, 52K
