# Supplementary material for: A Retrospective Cohort Analysis Shows that Coadministration of Minocycline with Colistin in Critically Ill Patients Is Associated with Reduced Frequency of Acute Renal Failure
Source: Antimicrob Agents Chemother. 2017 Dec 21;62(1):e01165-17. doi: 10.1128/AAC.01165-17 (PMC5740356; doi:10.1128/AAC.01165-17)
Supplement: Supplemental material [file AAC.01165-17_zac001186749s1.pdf]

SUPPLEMENTARY Table 1. Other Medications Associated with Acute Renal Failure

ACICLOVIR SODIUM	ISEPAMICIN
ADEFOVIR DIPIVOXIL	ISEPAMICIN SULFATE
AMIKACIN	LITHIUM
AMPHOTERICIN B	LITHIUM CARBONATE
AMPHOTERICINE B, LIPOSOME	METHOTREXATE
CARBOPLATIN	METICILLIN
CELECOXIB	MICRONOMICIN
CYCLOSPORIN	NETILMICIN
CONTRAST MEDIA, IV contrast dye	NETILMICIN SULFATE
CYCLOPHOSPHAMIDE	NOREPINEPHRINE
DIAGNOSTIC RADIOPHARMACEUTICALS	NOREPINEPHRINE BITARTRATE
DOPAMINE	PAROMOMYCIN
DOPAMINE HYDROCHLORIDE	STREPTOMYCIN
EPINEPHRINE	TACROLIMUS
GENTAMICIN	TENOFOVIR
INDINAVIR SULFATE	TENOFOVIR DISOPROXIL FUMARATE
IODIXANOL	TOBRAMYCIN
IOHEXOL	TOBRAMYCIN SULFATE
IOPROMIDE	VANCOMYCIN
FUROSEMIDE	

SUPPLEMENTARY Table 2. Concomitant Medications That Might Cause Acute Renal Failure by Population

	**Primary Population**			**PSM Population**		
**Variable**	**CST (N=4,817)**	**CST-MIN (N=93)**	***P* value**	**CST (N=664)**	**CST-MIN (N=83)**	**Absolute Standard Difference (*P*-value)**
All medications (listed in Supplemental Table 1)	98.2%	98.9%	1.000	99.1%	98.8%	2.9% (0.563)
Aminoglycoside	51.7%	79.6%	<0.001	71.4%	77.1%	13.0% (0.274)
Amikacin	16.4%	21.5%	0.185	18.1%	19.2%	3.1% (0.789)
Gentamicin	17.0%	9.7%	0.063	12.8%	10.8%	6.1% (0.612)
Tobramycin	30.3%	66.7%	<0.001	58.4%	62.6%	8.6% (0.462)

Contrast medias	11.1%	33.3%	<0.001	23.3%	27.7%	10.0%<br>(0.379)
Vancomycin	86.5%	89.2%	0.446	86.7%	87.9%	3.6%<br>(0.759)
Furosemide	66.2%	64.5%	0.730	65.2%	66.3%	2.2%<br>(0.849)
Others	69.0%	68.8%	0.976	68.1%	68.7%	1.3%<br>(0.912)

SUPPLEMENTARY Table 3. CST Dosing by CRD status

**Variable**	**CST (N=4,817)**	**CST-MIN (N=93)**	***P* value**
Patients with baseline CRD			
Days on CST			
Mean±SD	9.6±8.5	13.7±11.7	**0.004**
Median (Q1, Q3)	7 (5, 12)	10 (7, 18)	
Total vials of CST			
Mean±SD	17.7±18.5	21.6±17.3	0.060
Median (Q1, Q3)	12 (7, 22)	16 (9, 29)	
Daily vials of CST*			
1 vial	38.6%	48.9%	0.863
2 vials	46.2%	38.3%	
3 vials	11.0%	10.6%	
>=4 vials	4.1%	2.1%	
Patients without baseline CRD			
Days on CST			
Mean±SD	10.2±8.2	11.6±9.0	0.349

Median (Q1, Q3)	8 (5, 13)	9 (6, 15)	
Total vials of CST			
Mean±SD	23.6±25.4	24.1±20.6	0.951
Median (Q1, Q3)	17 (9, 29)	17 (9, 36)	
Daily vials of CST*			
1 vial	12.6%	21.7%	0.288
2 vials	58.7%	65.2%	
3 vials	18.4%	8.7%	
>=4 vials	10.3%	4.3%	

*1 vial colistin = 150mg colistin base activity (CBA)
